# Assessing the quality of life among Pakistani general population and their associated factors by using the World Health Organization’s quality of life instrument (WHOQOL-BREF): a population based cross-sectional study

**DOI:** 10.1186/s12955-018-1065-x

**Published:** 2019-01-14

**Authors:** Fahad Saqib Lodhi, Ali Montazeri, Saharnaz Nedjat, Mahmoud Mahmoodi, Umer Farooq, Mehdi Yaseri, Amir Kasaeian, Kourosh Holakouie-Naieni

**Affiliations:** 1 Department of Epidemiology and Biostatistics, School of Public Health, Tehran University of Medical Sciences, Tehran, Iran; 2 Academic Center for Education, Culture and Research, Institute for health Sciences Research, Tehran, Iran; 3 Community Medicine Department, Ayub Medical College, Abbottabad, Pakistan; 4 Non-communicable Diseases Research Center, Tehran University of Medical Sciences, Tehran, Iran; 5 Hematology-Oncology and Stem Cell Transplantation Research Center, Tehran University of Medical Sciences, Tehran, Iran

**Keywords:** Quality of life (QOL), WHOQOL-BREF, General population, Pakistan

## Abstract

**Background:**

Measuring quality of life (QOL) in a population is important for the predictions of health and social care needs. In Pakistan, health related quality of life data exist but there are no quality of life data of general population. In this study, quality of life was assessed among the Pakistani general population and their associated factors by using the World Health Organization’s quality of life instrument (WHOQOL-BREF).

**Methodology:**

A population-based cross-sectional study was carried out in all 52 Union Councils of District Abbottabad, Khaber Pkutunkhua province, Pakistan from March 2015 to August 2015. Multi-stage cluster sampling technique was employed in this study. Quality of life was measured by using the validated WHOQOL-BREF instrument, along with socioeconomic, demographic, and World Bank social capital questions in this population- based study. The data were collected through households, utilizing face to face interviews. The association between socio-demographic variables and quality of life domains were determined by using both univariate and multivariate analysis. Descriptive statistics were derived, and a multilevel linear regression using backward analysis allowing to obtain final model for each domain was achieved to recognize the variables that affect quality of life score.

**Results:**

A total of 2063 participants were included in this study (51.2% male, 48.2% female). Mean age of participants was 37.9, SD = 13.2; ranging from 18 to 90. Mean score of quality of life domains (physical, psychological, social relationship and environmental domains) were 65.0 (SD = 15.2), 67.4 (SD = 15.0), 72.0 (SD = 16.5), 55.5 (SD = 15.0), respectively. Overall, socioeconomic status was established to be the strongest predictor of poorer quality of life for all domains as a change in SES from high to low results in reduction about (β = − 5.85, β = − 9.03, β = − 8.33, β = − 9.98, *p* < 0.001). Similarly, type of residency was negatively associated with physical, psychological and environmental domains while age and sex were negatively associated with physical, psychological and relationship domains in final model. Furthermore social capital (β = 0.09, β = 0.13, β =0.14, β =0.15, *p* < 0.001) had a positive effect on Pakistani quality of life. Overall, subjective quality of life was found to be low in our population and extremely varied by socio-demographic variables.

**Conclusions:**

Increasing age, having average and lower socioeconomic status and living in the rural area were found to be the strong predictor of poorer quality of life in all domains, while total social capital score had a positive effect on Pakistani quality of life scores.

**Electronic supplementary material:**

The online version of this article (10.1186/s12955-018-1065-x) contains supplementary material, which is available to authorized users.

## Introduction

Health is defined by World Health organization (WHO) as” state of complete physical, mental and social well-being, not merely the absence of disease or infirmity” [1]. It is believed that the healthy individual enjoys the satisfactory quality of life level. In recent years, the concept of quality of life has attracted significant attention in public health researchers. More interest has been shown by scholars to measure and assess the quality of life of the general population that has become a major outcome measure in health-related research all over the world [2–4].

Quality of life is an extensive approach that could be explained in multiple ways, but there is an appreciable consensus between the quality of life researchers that quality of life is multi-dimensional and can be evaluated from subjective as well as objective perspectives [5, 6]. The World Health Organization (WHO) defines quality of life, as a person’s approach, their position in life, in the background of their culture and value system, they inhabit in relation to expectancies, patterns, and concerns [5]. Quality of life can be measured by different instruments both generic and disease specific. WHO-QOL is a generic instrument to measure quality of life. WHOQOL-BREF is a known and acceptable instrument for cross-cultural comparison and available in more than 40 countries. Instrument validity has been accepted by appraising the subjective quality of life of the general public. The Pakistani version of WHOQOL-BREF has been recognized to be valid and reliable in the assessment of quality of life in Pakistani individuals [7].

The quality of life is affected by multiple factors depending upon cultures. Previous research showed factors such as age, gender, marital status, education, place of living, health status, employment, and socioeconomic status. These were generally studied in quality of life research and most of them are associated to quality of life [2, 5, 8, 9]. Social capital is one of the important determinants affecting the quality of life of the populations. Improving the level of people’s social capital is an equal situation for other effective variables that can lead to the remarkable improvement in people’s quality of life [5, 6].

This study reports specific information on quality of life predictors of a sample of Pakistani general population. To the best of our knowledge, there has been no population-based study conducted in Pakistan which assessed all the domains of quality of life and its associated factors. This study aimed to identify the quality of life among Pakistani general population and their associated factors by using the World Health Organization’s quality of life instrument (WHOQOL-BREF).

## Methods

### Study design

This was a population-based cross-sectional study carried out in all 52 Union Councils of District Abbottabad, Khyber Pakhtunkhwa province, Pakistan, from March 2015 to August 2015. The district is divided into two Tehsils Abbottabad and Hevellian comprising 52 Union Councils. Area-wise, District Abbottabad is 1967 sq. km, with a population of 881,000 and an average annual growth rate of 1.82%. The average size of each household is calculated as 4 to 6 individuals [10].

### Sample size

A sample of at least 1936 participants was required with the difference of 2.5 QOL scores between joint and nuclear family system, for QOL scores to be considered sufficient of practical significance. We calculated the sample size by keeping persons in both joint and nuclear group, having 90% power of the study with 95% confidence interval, 1.8 design effect and 10% missing response rate.

### Recruitment

In this study, participants were randomly selected from both nuclear and joint family houses from all 52 union council of District Abbottabad, Pakistan. The Following criteria were used for selection: (1) Age 18 years and above (2), absence of any apparent or diagnosed mental illness (3), and permanent resident of union council for at least 5 years. Guests and temporary residents were excluded from the study.

**Fig. 1:**
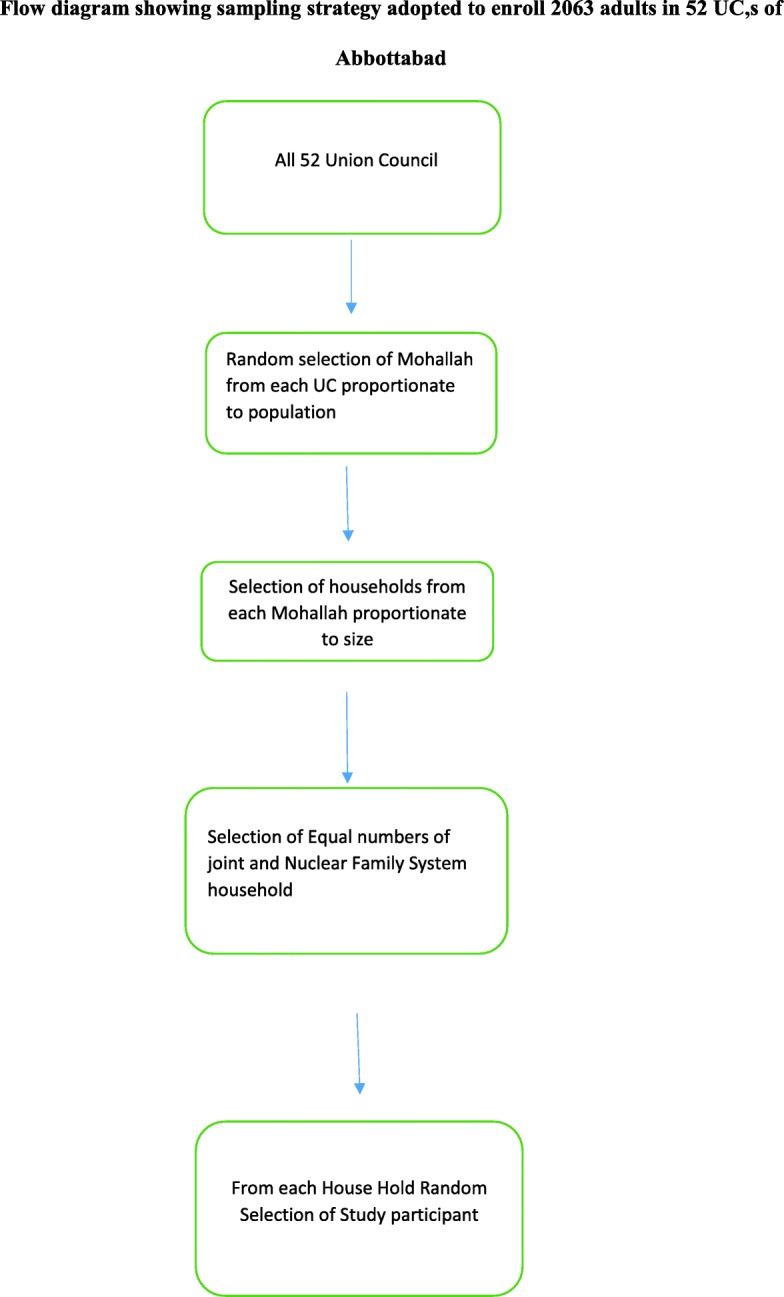
Flow diagram showing sampling strategy adopted to enroll 2063 adults in 52 UC, s of Abbottabad

Multi-stage cluster sampling technique was employed in this study. District Abbottabad consists of 52 union councils (UCs). All union councils were included in the study. Each union council was further divided into several more blocks in the shape of Mohallah (area of town or village where people live and communicate with each other). We did proportionate sampling according to the 1998 census population [11] of UCs to select the Mohallah for the next stage. In the first step, we randomly selected these blocks (Mohallah) using simple random sampling technique. In the next stage, we selected the number of households in that selected block using a random sampling technique again. In each union council, the size of both types of households was proportional to the population size of that union council (Additional file 1). After selecting the first house as the index of the block by random sampling, houses on the right side of the index house were selected to fulfill the computed sample size in each union council. A simple random sampling technique was used for the selection of person (≥18 years) from each house and if the randomly selected person was not present at home at the time of the interview (Fig. 1), the interviewer visited that house again and conducted the interview. In case of not being able to contact the person after three visits, the next randomly selected household was approached for the interview. A 1 day training session was conducted for administering the questionnaire prior to data collection for lady health workers of all UCs by the principal investigator. Questionnaires were administered by trained lady health workers of each union council through a face to face interview. All the interviews were conducted in a separate room or place which were separated from other family members to guarantee complete privacy and confidentiality.

### Data collection

#### Data collection tools were as follows

1. A demographic questionnaire included variables such as age, gender, marital status (married, widowed, divorced, separated, and never married), Type of family (joint and nuclear), Residence type (urban and rural), Residence ownership (owner, not owner), Respondent education (no education, madressa, can read/write, primary- up to grade 5, secondary education-up to grade 12 and tertiary-up to grade 16 or above), Occupational status (employed, unemployed and retired) and any disease/disability in the family.
2. The Socioeconomic characteristics were assessed by taking household conditions, sources of drinking water, sanitation facilities, availability of electricity, housing facilities, possession of durable goods, means of transport, inventory of household and personal items such as chairs, clocks, buckets, radios, television sets, fans, stoves or cookers, cars, and telephones. This list was composed of 21 such items used in Pakistan demographic and health survey in 2013 [12]. The Wealth index was measured by an index established from principal component analysis of objects representing ownership of household durables and residence characteristics.
3. WHOQOL-BREF is a generic instrument to measure the subjective QOL. WHO started a project in 1991 and the research product of 15 participating countries were used to finalize the instrument. The instrument has 100 questions and is used with people having different diseases, illness conditions and cultural subgroups [6]. The WHOQOL-BREF is the shorter version having 26 questions as compared to 100, and it covers a wide range of quality of life QOL items divided into four domains: Physical (7 questions), Psychological (6 questions), Social relationship (3 questions) and Environmental health domain (8 questions) [13]. The last two items are about general health and overall quality of life. Each item score from 1 to 5 on a Likert scale where 1 represents (very dissatisfied/very poor) and 5 represents (Very satisfied). The score is then transformed into a linear scale between 0 to 100 scales, where 0 being the minimum satisfactory and 100 being the maximum favorite [14].
  For the present study, the WHOQOL-BREF questionnaire was first translated into the national Urdu language and its validation was prepared by the Professionals of the concerned field- Sociologists and by Medical doctors. Reliability of the four dimensions [Physical, Psychological, Environmental and Relationship] met the minimum Reliability standards of Cronbach’s alpha that is .78, .71, .73 and .56, respectively [7].
4. World Bank Social Capital Questioners (SC-IQ). For the developing countries, World Bank Social Capital Questioners (SC-IQ) was used to study social capital among families. SC-IQ consists of 6 domains and included 27 questions [15]. With the help of country experts, we extracted five questions from the core questioner with having Cronbach’s alpha 0.64. These five selected questions are as follows: 1)overall trust, 2)trust in local Government, 3)trust in central Government, 4)community cooperation, 5)safety at home. Each question is in the form of a Likert scale. The score of each question ranges from 0 to 100 and higher scores indicate an improved condition.

### Statistical analysis

The statistical analysis software Stata version 13.0 (Stata Corp, College Station, TX, USA) was used for the analysis of data. To analyze, descriptive statistics such as frequency table, mean, standard deviation, inter-quartile range (IQR) and range were used. Comparison of the sub group when considering the confounding effect was performed by regression methods (linear regression). Univariate and adjusted analysis were undertaken in which domain scores were measured as dependent and variable for other socio-demographics were included as independent variables. Separate univariate models explored each of the variables alone with the outcome.

To develop the final model, we first ran full model which included all the variables. Then in final model, using stepwise backward approach, we eliminated variables with a *p* value > 0.05.

In all calculations design effect was considered. Random effect of union council and cluster level were further examined in the multilevel-multivariate analysis.

### Multilevel regression analysis of multilevel cluster data

Multilevel models are increasingly used to understand the contribution of sources of variation at different levels [16]. A two –level continuous random intercept model with individuals nested within clusters was applied to explore the variability explained by individuals and cluster level variables taking the correlated nature of data into account [17]. Such multilevel model allows the estimation of (a) the conditional relationship between QOL score and individual predictors (fixed effect parameters), and (b) variation between clusters that cannot be explained by individual predictors (random effect parameters).

## Results

### Socio-Demographic characteristics

The participant characteristics and QOL score domains are presented in Table 1.We recruited 2063 (1058 male and 1005 female) study participants for our study in district Abbottabad. The refusal rate was 2.5%. Among all participants subjects, 51% were from joint family and 49% from nuclear family subjects. The mean age of participants was 37.9 (SD = 13.2) years (ranging from 18 to 90). Most families were living in their own houses 77.5% and families living in the rural area were 71%. 79.4% of the participants were married. The majority of the study participants were from middle-class families at 33.4. 39.3% participants were not working/unemployed, 31.8% had an education up to grade 12.

**Table 1:** The characteristics of the study sample and Mean scores of quality of life domains among different subgroups, Abbottabad, Pakistan 2016 (*n* = 2063)

Variable		Domains of Quality of life (mean ± SD)				
	Frequency (%)	Physical (2063)	Psychological (2063)	Relationship (2063)	Environmental (2063)	General facet
Type of family						
Joint	1053 (51.04%)	65.0 ± 15.2	67.9 ± 14.3	72.4 ± 16.8	56.6 ± 14.3	69.2 ± 16.6
Nuclear	1010(48.96)	64.8 ± 15.2	67.0 ± 15.3	71.4 ± 16.0	54.3 ± 15.1	66.9 ± 18.5
Type of Residence						
Urban	598(29%)	68.0 ± 15.7	71.0 ± 14.4	74.09 ± 15.8	59.9 ± 14.2	71.0 ± 16.5
Rural	1465(71%)	63.7 ± 14.8	65.9 ± 14.7	71.01 ± 16.6	53.7 ± 14.5	67.0 ± 17.9
Sex						
Male	1058 (51.2%)	66.4 ± 15.5	68.7 ± 14.7	72.4 ± 15.8	56.0 ± 14.48	69.7 ± 16.7
Female	1005(48.7%)	63.5 ± 14.7	66.0 ± 15.1	71.3 ± 17.0	54.8 ± 15.0	66.4 ± 18.3
Age						
<30	720(34.9%)	67.7 ± 14.5	68.38 ± 14.0	71.99 ± 16.1	55.84 ± 14.6	69.7 ± 16.5
31–40	636(30.8%)	64.6 ± 14.2	67.16 ± 15.1	72.27 ± 16.2	54.57 ± 14.6	68.0 ± 17.8
41–50	389(18.8%)	64.0 ± 15.4	67.96 ± 14.7	72.38 ± 16.4	55.98 ± 14.5	67.1 ± 18.0
>50	318(15.4%)	60.38 ± 16.9	64.91 ± 15.8	70.16 ± 17.7	55.52 ± 15.5	65.8 ± 18.8
Residence Ownership						
Owner	1599(77.5%)	64.9 ± 15.2	67.3 ± 14.6	71.9 ± 16.4	55.5 ± 14.7	68.3 ± 17.2
Not Owner	464 (22.5%)	65.6 ± 15.1	68.6 ± 15.4	71.5 ± 16.6	55.5 ± 14.9	69.6 ± 18.5
Marital Status						
Married	1639 (79.4%)	64.2 ± 14.8	67.4 ± 14.7	72.8 ± 16.1	55.5 ± 14.6	68.3 ± 17.4
Widow	60 (2.9%)	55.0 ± 17.1	55.9 ± 18.2	61.8 ± 20.6	46.1 ± 14.2	54.3 ± 23.2
Divorced	6 (2.0%)	76.8 ± 10.0	75 ± 9.1	62.5 ± 15.5	59.9 ± 13.6	70.8 ± 12.9
Separated	9 (0.4%)	70.9 ± 16.1	69.0 ± 12.6	54.8 ± 28.8	54.5 ± 9.1	71.4 ± 17.2
Never married	349(16.9%)	69.8 ± 15.1	68.9 ± 14.2	70.0 ± 15.9	56.6 ± 15.0	69.6 ± 16.3
Education						
No education	322(15.6%)	59.0 ± 15.5	61.5 ± 16.5	67.3 ± 17.8	51.4 ± 15.6	60.1 ± 20.8
Madressa	47(2.2%)	63.9 ± 18.2	68.6 ± 14.3	73.9 ± 12.2	58.3 ± 14.9	70.8 ± 15.0
Can read/write	211(10.2%)	61.8 ± 15.8	63.9 ± 15.5	68.8 ± 17.5	54.0 ± 14.1	64.8 ± 17.5
Primary	637(30.8%)	65.7 ± 14.6	67.7 ± 13.9	73.0 ± 15.9	56.3 ± 13.9	68.5 ± 16.8
Secondary	658(31.8%)	67.8 ± 14.8	70.5 ± 14.0	73.1 ± 15.9	56.7 ± 14.9	71.2 ± 16.1
Tertiary	188(9.1%)	66.3 ± 13.3	69.3 ± 13.3	74.8 ± 15.4	56.4 ± 14.7	73.2 ± 14.3
Disease						
Physical disability	28(1.4%)	57.5 ± 19.4	62.0 ± 17.3	71.1 ± 10.2	53.8 ± 13.6	63.3 ± 19.2
Hypertension	154(7.5%)	61.3 ± 16.6	66.0 ± 15.6	70.7 ± 16.6	54.8 ± 16.7	66.1 ± 17.7
Diabetes	58(2.8%)	55.4 ± 17.7	62.0 ± 17.6	69.3 ± 19.0	52.9 ± 15.8	60.9 ± 21.8
Other	980(47.5%)	63.7 ± 15.1	66.2 ± 14.8	71.2 ± 16.5	54.6 ± 14.8	67.0 ± 18.3
None	843(41%)	68.0 ± 13.8	69.7 ± 14.0	73.2 ± 16.2	56.8 ± 14.1	70.5 ± 15.9
Employment status						
Working	1162(56.3%)	66.3 ± 14.2	68.8 ± 13.9	72.3 ± 16.3	55.9 ± 14.4	70.1 ± 16.3
Not Working	810(39.3%)	63.1 ± 15.9	65.6 ± 15.8	71.2 ± 16.6	54.6 ± 15.1	65.5 ± 18.8
Retired	91(4.4%)	63.0 ± 18.3	65.9 ± 15.3	73.1 ± 16.6	57.5 ± 14.8	66.5 ± 18.4
Socioeconomic status						
High	687(33.3%)	68.2 ± 14.6	71.8 ± 13.7	75.5 ± 15.3	60.8 ± 13.8	74.4 ± 15.5
Average	690(33.4%)	65.4 ± 14.9	68.6 ± 13.5	73.5 ± 13.5	56.4 ± 13.5	68.7 ± 15.6
Low	686(33.3%)	61.1 ± 15.2	63.2 ± 15.3	66.7 ± 15.3	49.3 ± 14.7	61.3 ± 19.0
Social Capital						
High SC	278(13.5%)	66.6 ± 14.6	69.7 ± 13.6	75.1 ± 15.7	58.8 ± 13.8	71.8 ± 16.7
Moderate SC	1571(76.1%)	65.2 ± 15.1	67.7 ± 14.7	72.1 ± 16.1	55.5 ± 14.4	68.3 ± 17.2
Low SC	214(10.4%)	61.3 ± 16.2	63.7 ± 16.2	66.7 ± 18.2	50.9 ± 16.6	62.2 ± 19.5
All	2063	65.0 ± 15.2	67.4 ± 15.0	72.0 ± 16.5	55.5 ± 15.0	68.0 ± 18.0

Using the 0–100% scoring method: Mean (SD) [95% Confidence Intervals]. Values are mean ± standard deviation

154 (7.5%), 58 (2.8%), and 28(1.4%) were suffering from hypertension, diabetes and physical disability respectively. Half of the study population (47.5%) were suffering from other minor health issues.

### Quality of life scores in each domain

As presented in Table 1, the mean scores for physical, psychological, social relationship, and environmental domains were 65.0 (SD = 15.2), 67.4 (SD = 15.0), 72.0(SD = 16.5), 55.5 (SD = 15.0), respectively. General facet domain mean score was 68.0 (SD = 18.0).

Tables 2, 3, 4 and 5 show the association between different demographic, socioeconomic and total social capital score with domains of Quality of life through the univariate analysis and multilevel linear regression.

**Table 2:** Univariate and Multilevel Linear Regression showing association between socio demographic variables with Physical domain of QOL BREF in Abbottabad, Pakistan

Variable	Univariate analysis				Adjusted analysis			
	Beta coefficient	95% CI		*p* - value	Beta coefficient	95% CI		*p* - value
		LL	UL			LL	UL	
*Fixed – effect parameters*								
Type of family								
Joint	R^a^				R			0.999
Nuclear	−0.28	− 1.60	1.03	0.672	0.00	− 1.20	1.20	
Type of Residence								
Urban	R				R			0.029
Rural	−4.31	−5.76	− 2.87	<0.001	− 2.98	− 5.67	− 2.98	
Sex								
Male	R				R			<0.001
Female	−2.90	−4.17	−1.62	<0.001	− 2.39	− 3.80	−0.99	
Residence Ownership								
Owner	R				R			0.69
Not owner	0.71	−1.51	2.94	0.528	−0.46	−2.66	1.75	
Age (decade)	−0.23	−0.28	−1.8	<0.001	− 0.18	−.23	−.11	<0.001
Marital Status								
Married	R				R			0.007
Widow	− 9.1	−13.04	−5.34	<0.001	−2.79	− 6.45	0.87	
Divorced	12.53	0.56	24.51	0.040	10.97	0.17	21.75	
Separated	6.67	−4.42	17.77	0.239	2.74	−7.24	12.74	
Never married	5.50	3.78	7.23	<0.001	2.67	0.84	4.49	
Education								
No Education	−7.34	−10.02	−4.66	<0.001	−2.04	−4.82	0.74	0.010
Madressa	− 2.37	−7.22	2.48	0.339	−0.38	−4.96	4.21	
Can read / write	−4.50	−7.43	−1.57	0.003	−1.20	−4.09	1.69	
Primary	−0.59	−3.02	1.83	0.630	0.91	−1.45	3.28	
Secondary	1.44	−0.97	3.86	0.242	1.51	−0.79	3.81	
Tertiary	R				R			
Disease								
Physical disability	−10.54	−16.15	−4.93	<0.001	−9.20	−14.34	−4.08	<0.001
Hypertension	−6.78	−9.31	−4.19	<0.001	− 4.81	−7.51	−2.12	
Diabetes	−12.64	− 16.61	−8.68	<0.001	− 10.31	− 14.16	− 6.46	
Other	−4.37	−5.75	−3.00	<0.001	−4.55	−6.29	−2.80	
None	R				R			
Employment status								
Working	R				R			0.056
Not Working	−3.23	−4.59	−1.88	<0.001	− 1.75	− 3.21	−.28	
Retired	−3.35	−6.60	− 0.15	0.04	−1.38	− 3.21	1.60	
Socioeconomic status								
High	R				R			<0.001
Average	−2.82	−4.40	−1.24	<0.001	−1.71	−3.26	−0.17	
Low	−7.15	−8.73	−5.57	<0.001	−5.10	− 6.82	−3.38	
Social capital	0.10	0.06	0.14	<0.001	0.09	0.05	0.13	<0.001
*Random – effect parameters*								
					Estimate	Std. Err	95% confidence interval	
							LL	UL
Level 1: Union council								
Sex	–				0.69	1.14	0.02	17.15
Age	–				0.04	0.03	0.01	0.21
Social Capital	–				0.05	0.01	0.02	0.09
Constant	–				2.10	1.47	12.67	13.52
Level 2: Cluster number								
Constant	–				3.54	0.51	2.66	4.70
Residual	–				13.09	0.21	12.67	13.52

^*a*^*R* ., reference

*CI*, confidence interval

Adjusted analysis performed with all variables entered simultaneously into the model

Multiple linear regression was used for analysis and *p*-value <0.05 was considered statistically significant

**Table 3:** Univariate and Multilevel Linear Regression showing association between socio demographic variables with Psychological domain of QOL BREF in Abbottabad, Pakistan

Variable	Univariate analysis				Adjusted analysis			
	Beta coefficient	95% CI		*p* - value	Beta coefficient	95% CI		*p* - value
		LL	UL			LL	UL	
Fixed – effect parameters								
Type of family								
Joint	R^a^				R			0.828
Nuclear	−0.90	− 2.18	0.38	0.168	−0.12	− 1.29	1.03	
Type of Residence								
Urban	R				R			0.033
Rural	−5.08	−6.47	− 3.68	<0.001	−2.79	− 5.34	−0.22	
Sex								
Male	R				R			0.032
Female	−2.76	−4.05	−1.47	<0.001	− 1.51	− 2.89	−0.13	
Residence Ownership								
Owner	R				R			0.980
Not owner	1.32	−0.84	3.50	0.231	−0.02	−2.15	2.10	
Age (decade)	−0.11	−0.16	− 0.06	<0.001	− 0.08	−0.14	− 0.02	0.004
Marital Status								
Married	R				R			0.030
Widow	−11.52	−15.30	−7.731	<0.001	−5.51	−9.04	− 1.98	
Divorced	7.50	.4.28	19.29	0.212	6.30	−4.09	16.71	
Separated	1.55	−9.36	12.47	0.780	−1.51	−11.13	8.10	
Never married	1.48	−0.21	3.18	0.087	0.18	−1.57	1.94	
Education								
No Education	−7.80	−10.42	−5.19	<0.001	−2.84	−5.52	−0.16	<0.001
Madressa	−0.71	−5.43	4.00	0.767	1.51	−2.90	5.94	
Can read/write	−5.38	−8.23	−2.53	<0.001	−1.29	−4.09	1.49	
Primary	−1.56	−3.92	0.800	0.195	0.00	−2.29	2.29	
Secondary	1.20	−1.14	3.56	0.314	1.57	−.66	3.79	
Tertiary	R				R			
Disease								
Physical disability	−7.67	−13.21	−2.13	0.007	−6.34	−11.29	−1.39	<0.001
Hypertension	−3.71	−6.23	−1.18	0.004	−2.37	−4.97	0.22	
Diabetes	−7.66	−11.57	−3.74	<0.001	−5.45	−9.17	−1.73	
Other	−3.51	−4.87	−2.16	<0.001	−3.29	−4.99	−1.59	
None	R				R			
Employment status								
Working	R				R			0.013
Not Working	−3.19	−4.52	−1.88	<0.001	−2.12	− 3.53	−0.70	
Retired	−2.90	−6.06	0.27	0.073	−1.20	−4.09	1.67	
Socioeconomic status								
High	R				R			< 0.001
Average	−3.22	−4.72	−1.71	<0.001	−2.43	− 3.92	− 0.94	
Low	−10.04	−11.55	−8.53	<0.001	−7.84	−9.51	−6.19	
Social capital	0.14	0.10	0.19	<0.001	0.12	0.084	0.17	<0.001
*Random – effect parameters*								
					Estimate	Std. Err	95% confidence interval	
							LL	UL
Level 1: Union council								
Sex	–				1.08	0.69	0.30	3.82
Age	–				0.02	0.06	0.00	5.61
Social Capital	–				0.06	0.01	0.03	0.09
Constant	–				0.72	4.32	5.57	9.22
Level 2: Cluster number								
Constant	–				3.94	0.49	3.08	5.03
Residual	–				12.59	0.21	12.18	13.00

^a^*R* ., reference

*CI* confidence interval

Adjusted analysis performed with all variables entered simultaneously into the model

Multiple linear regression was used for analysis and *p*-value <0.05 was considered statistically significant

**Table 4:** Univariate and Multilevel Linear Regression showing association between socio demographic variables with Relationship domain of QOL BREF in Abbottabad, Pakistan

Variable	Univariate analysis				Adjusted analysis			
	Beta coefficient	95% CI		*p* - value	Beta coefficient	95% CI		*p* - value
		LL	UL			LL	UL	
Fixed – effect parameters								
Type of family								
Joint	R^a^				R			0.880
Nuclear	−.94	− 2.36	0.48	0.195	−.10	−1.44	1.23	
Type of Residence								
Urban	R				R			0.113
Rural	−3.08	−4.64	− 1.52	<0.001	− 2.14	− 4.79	0.50	
Sex								
Male	R				R			0.156
Female	−1.39	−2.82	.042	0.057	−1.12	−2.69	0.43	
Residence Ownership								
Owner	R				R			0.309
Not owner	−0.45	−2.87	1.97	0.714	−1.26	−3.70	1.17	
Age (decade)	−0.05	−0.10	0.001	0.077	− 0.91	−0.16	− 0.24	0.007
Marital Status								
Married	R				R			<0.001
Widow	−11.99	−15.19	−6.79	<0.001	−6.44	10.51	−2.38	
Divorced	−10.29	− 23.37	2.78	0.122	−8.84	20.79	3.10	
Separated	−18.03	−30.14	−5.93	0.003	−19.85	30.91	−8.79	
Never married	−2.87	−4.76	−.99	0.003	−4.45	−6.47	−2.44	
Education								
No Education	−7.43	−10.37	−4.49	<0.001	− 4.06	−7.14	−.98	0.012
Madressa	−.93	−6.23	4.37	0.730	1.12	−3.94	6.19	
Can read / write	−6.06	−9.27	−2.86	<0.001	− 2.82	−6.02	.37	
Primary	−1.78	−4.44	.87	0.186	−.403	−3.02	2.21	
Secondary	−1.73	−4.38	.91	0.198	−1.20	−3.76	1.33	
Tertiary	R				R			
Disease								
Physical disability	−2.01	−8.29	4.09	0.506	−1.46	−7.14	4.22	0.887
Hypertension	−2.55	−5.38	0.26	0.076	−1.02	−4.00	1.94	
Diabetes	−3.97	−8.34	0.39	0.075	−1.26	−5.52	3.00	
Other	−2.05	−3.57	−0.54	0.008	−0.86	−2.72	0.99	
None	R				R			
Employment status								
Working	R				R			0.295
Not Working	−1.14	−2.62	.33	0.12	0.53	−1.09	2.16	
Retired	0.72	−2.80	4.26	0.69	2.53	−.77	5.83	
Socioeconomic status								
High	R				R			<0.001
Average	−2.05	−3.74	−0.35	0.018	−1.87	−3.58	−0.15	
Low	−8.84	−10.54	−7.14	<0.001	−7.19	−9.00	− 5.28	
Social capital	0.15	0.02	6.73	<0.001	0.12	0.09	0.17	<0.001
*Random – effect parameters*								
					Estimate	Std. Err	95% confidence interval	
							LL	UL
Level 1: Union council								
Sex	–				0.71	1.02	0.04	11.90
Age	–				0.04	0.03	0.01	0.17
Social Capital	–				0.02	0.03	0.001	0.390
Constant	–				0.000	0.002	1.14	0.363
Level 2: Cluster number								
Constant	–				5.13	0.56	4.15	6.34
Residual	–				14.46	0.24	14.00	14.94

^a^*R* ., reference

*CI* confidence interval

Adjusted analysis performed with all variables entered simultaneously into the model

Multiple linear regression was used for analysis and p-value <0.05 was considered statistically significa

**Table 5:** Univariate and Multilevel Linear Regression showing association between socio demographic variables with Environmental domain of QOL BREF in Abbottabad, Pakistan

Variable	Univariate analysis				Adjusted analysis			
	Beta coefficient	95% CI		*p* - value	Beta coefficient	95% CI		*p* - value
		LL	UL			LL	UL	
Fixed – effect parameters								
Type of family								
Joint	R^a^				R			0.073
Nuclear	− 2.21	−3.49	−.94	<0.001	−.99	− 2.09	0.093	
Type of Residence								
Urban	R				R			0.002
Rural	−6.27	−7.64	−4.89	<0.001	−5.28	−8.63	−1.94	
Sex								
Male	R				R			0.891
Female	−1.13	−2.44	.167	0.087	.09	−1.25	1.44	
Residence Ownership								
Owner	R				R			0.242
Not owner	.10	−2.06	2.27	0.924	−1.20	−3.21	.811	
Age (decade)	−0.1	−0.60	0.35	0.588	0.340	−0.22	0.91	0.239
Marital Status								
Married	R				R			<0.001
Widow	−9.40	−13.18	−5.62	<0.001	−7.04	− 10.37	−3.72	
Divorced	4.34	−7.40	16.10	0.468	4.33	−5.40	14.08	
Separated	−1.08	−11.96	9.80	0.845	−2.51	−11.54	6.51	
Never married	1.05	−.64	2.75	0.223	1.37	−.28	3.01	
Education								
No Education	−5.02	−7.66	−2.39	<0.001	−.75	−3.29	1.79	0.065
Madressa	1.86	−2.89	6.62	0.443	4.26	.10	8.42	
Can read / write	−2.44	−5.32	.43	0.096	.96	−1.68	3.59	
Primary	−0.06	−2.44	2.31	0.957	1.29	−0.87	3.44	
Secondary	0.28	−2.09	2.66	0.812	1.24	−0.84	3.33	
Tertiary	R				R			
Disease								
Physical disability	−3.02	−8.56	2.52	0.285	−1.96	−6.61	2.69	0.114
Hypertension	−1.97	−4.49	.561	0.127	−2.48	−4.99	.030	
Diabetes	−3.90	−7.82	.009	0.050	−3.80	−7.32	−.288	
Other	−2.20	−3.56	−.85	0.001	−1.61	−3.35	.12	
None	R				R			
Employment status								
Working	R				R			0.201
Not Working	−1.28	−2.61	.03	0.056	−.55	−1.88	.79	
Retired	1.55	−1.61	4.71	0.336	2.01	−.690	4.71	
Socioeconomic status								
High	R				R			<0.001
Average	−4.46	−5.95	−2.99	<0.001	−4.05	−5.46	−2.65	
Low	−11.58	−13.06	−10.09	<0.001	−9.20	− 10.79	−7.62	
Social capital	0.17	0.12	0.21	<0.001	.14	.10	.18	<0.001
*Random – effect parameters*								
					Estimate	Std. Err	95% confidence interval	
							LL	UL
Level 1: Union council								
Sex	–				1.67	0.69	0.74	3.73
Age	–				0.07	0.03	0.03	0.14
Social Capital	–				0.07	0.01	0.04	0.12
Constant	–				2.81	1.66	0.88	8.96
Level 2: Cluster number								
Constant	–				4.90	0.48	4.04	5.94
Residual	–				11.68	0.19	11.29	12.08

^a^ *R ., reference*

*CI* confidence interval;

Adjusted analysis performed with all variables entered simultaneously into the model

Multiple linear regression was used for analysis and p-value <0.05 was considered statistically significant

In univariate analysis of association between socio demographic variable with physical health domain, type of residence, sex, age, marital status, residence ownership, disease status, education status, employment, socio-economic status (SES) and social capital were significantly associated with the physical domain of quality of life. Psychological health domain was associated with type of residence, sex, age, marital status, no education, madrassa, disease status, education status, employment, SES and social capital were significantly associated with the psychological domain of quality of life. Social health domain was associated with type of residence, sex, marital status, no education, SES and social capital were significantly associated. Type of family system, type of residence, marital-status, lack of education, other health issues, unemployed status, SES and social capital were all significantly associated with environmental domain of QOL (*p* < 0.05).

In adjusted analysis, type of residence, sex, age, marital status, education, disease, socioeconomic status and social capital were significantly associated with physical health; Psychological health was associated with type of residence, sex, age, marital status, education, disease, employment status, socioeconomic status and social capital; Social relationship was associated with age, marital status, education, disease, socioeconomic status and social capital. Environmental domain was associated with type of residence, marital status, socioeconomic status and social capital.

Final Multiple linear regression model (Table 6).

**Table 6:** Final model after Multiple linear regression analysis for physical, psychological, social and environmental health domains of QOL BREF in Abbottabad, Pakistan

	Physical Domain				Psychological Domain				Relationship Domain				Environmental Domain			
	*Fixed – effect parameters*															
Variable	β	*P* value	95% CI		β	*P* value	95% CI		β	*P* value	95% CI		β	*P* value	95% CI	
			LL	UL			LL	UL			LL	UL			LL	UL
Type of Residence																
Urban	R				R				R				R			
Rural	−3.19	0.017	−5.81	−0.57	−2.70	0.036	−5.23	−0.18	–	–	–	–	−5.34	0.002	−8.66	−2.02
Sex																
Male	R				R				R				R			
Female	−3.24	<0.001	−4.59	−1.88	−3.25	<0.001	−4.48	−2.01	−1.61	0.024	−3.01	−0.21	–	–	–	–
Age (decade)	−0.24	<0.001	−0.29	− 0.19	− 0.123	<0.001	− 0.17	−0.07	− 0.07	0.018	− 0.12	−0.01	–	–	–	–
Disease																
Physical disability	−9.27	<0.001	−14.4	−4.11	−6.55	0.010	−11.5	− 1.55	–	–	–	–	–	–	–	–
Hypertension	−4.42	<0.001	−7.11	−1.73	−2.56	0.054	−5.17	0.05	–	–	–	–	–	–	–	–
Diabetes	−10.5	<0.001	−14.4	−6.64	−5.78	0.003	−9.53	−2.03	–	–	–	–	–	–	–	–
Other	−4.61	<0.001	−6.37	−2.86	−3.59	<0.001	−5.29	−1.88	–	–	–	–	–	–	–	–
None	R				R				R				R			
Socioeconomic status																
High	R				R				R				R			
Average	−2.13	0.006	−3.66	−0.60	−2.99	<0.001	−4.47	−1.51	− 2.24	0.009	− 3.92	− 0.55	− 4.31	<0.001	−5.69	− 2.92
Low	−5.85	<0.001	−7.50	− 4.20	−9.03	<0.001	−10.63	−7.44	−8.33	<0.001	− 10.1	−6.54	− 9.98	<0.001	−11.5	−8.47
Social capital	0.09	<0.001	0.06	0.18	0.13	<0.001	0.09	0.17	0.14	<0.001	0.09	0.18	0.15	<0.001	0.11	0.19
	*Random – effect parameters*															
	Est	SE	95% CI		Est	SE	95% CI		Est	SE	95% CI		Est	SE	95% CI	
			LL	UL			LL	UL			LL	UL			LL	UL
	Level 1: Union council															
Sex (sd)	0.20	1.27	1.1e^−6^	3.9e^4^	0.97	0.69	0.24	3.95	0.97	0.76	0.21	4.53	1.81	0.66	0.89	3.71
Age (sd)	0.03	0.04	0.004	0.27	0.03	0.03	0.004	0.26	0.03	0.04	0.003	0.36	0.07	0.02	0.04	0.14
Social Capital (sd)	0.05	0.02	0.02	0.09	0.06	0.01	0.04	0.09	0.02	0.03	0.003	0.24	0.07	0.02	0.04	0.12
Constant (sd)	2.27	1.27	0.76	6.80	0.004	0.01	1.4e^−5^	0.90	8.7e^−7^	3.1e^−6^	8.9e^−10^	8.5e^−4^	2.38	1.84	0.52	10.86
	Level 2: Cluster number															
Constant (sd)	3.63	0.51	2.76	4.79	3.95	0.49	3.09	5.05	5.39	0.54	4.42	6.56	5.03	0.48	4.16	6.08
Residual (sd)	13.2	0.22	12.77	13.63	12.74	0.21	12.33	13.16	14.65	0.24	14.19	15.14	11.81	0.20	11.42	12.21

^a^β,. Beta coefficient

*CI* confidence interval;

The Linear regression final model adjusted type of family, residence ownership, marital status, education and employment status in physical domain and Psychological domain. Type of family, Type of residence, residence ownership, marital status, education, employment status and disease for Social domain. Type of family, residence ownership, marital status, Age, sex, education, employment status, disease in Environmental domain. *P*-value <0.05 was considered statistically significant

The short dashes mean that the variable was removed by the stepwise deletion process in regression analysis

Table 6 demonstrates the results of the final multiple linear regression model. Variables significantly associated with the physical domain of quality of life included: residence was negatively associated with physical QOL scores and it was 3.19 unit reduction when changing from urban to rural. Females were found to have 3.24 units less QOL scores as compared to males. Age was negatively associated with QOL scores with each unit change would lead to 0.24 unit reduction. Presence of disease was significantly associated as scores declined with the presence of disease. Socio-economic status also had a significant association as a change in SES from high to low resulted in a reduction of approximately 5.85 units in scores. With each unit increase in score of social capital, there is 0.09 unit increase in scores.

The psychological domain was significantly and negatively associated with type of residence which was found to have a 2.7 unit reduction when changing from urban to rural in psychological QOL scores. Females were also found to have 3.25 units less scores as compared to males. Age was negatively associated with QOL scores with each unit change would lead to 0.123 unit reduction in QOL score. Presence of disease was significantly associated as scores declined with the presence of disease. Socio-economic status also had a significant negative association as a change in SES from high to low resulted in reduction of 9.03 units in scores. With each unit increase in score of social capital, there was a corresponding 0.13 unit increase.

Several Variables significantly associated with the social relationship domain of quality life included; Females were found to have 1.61 units less QOL scores as compared to males. Age was negatively associated with QOL scores with each unit change leading to a 0.07 unit reduction. Socio-economic status had a significant negative association as a change in SES from high to low result in the reduction of 8.33 units in scores. With each unit increase in score of social capital, there is a 0.14 unit increase.

Variables significantly associated with the environmental domain of quality of life included: Residence type was significantly negatively associated with environmental QOL scores and resulted in a 5.34 unit reduction when changing from urban to rural. A change in SES from high to low resulted in reduction of 9.98 units in scores. With each unit increase in score of social capital, there is a 0.15 unit increase in environmental QOL with significant *p*-value in the final adjusted model (*P* = < 0.001).

Overall, increasing age, having average or lower socioeconomic status and living in a rural area were found to be strong predictors of poorer quality of life in all domains, while total social capital score had a positive effect on Pakistani QOL scores.

## Discussion

The present study was performed to assess the vital information on subjective Quality of life (QOL) among Pakistani people. This study also provides us information regarding the effect of family structure on QOL domains and to explore out the relationship between the socio-demographic factors and all four domain of Quality of life. To the best of our knowledge, this is one of the first studies using WHOQOL-BREF measuring QOL among the Pakistani general population.

Our study indicated that overall, QOL scores were found to be low among Pakistani the general population. Compared with other countries, QOL in Pakistan was a bit higher than Iran in psychological, social relationship and environmental domains [3]. When compared with data from Brazil [2], the scores for Pakistan were lower for the psychological, social and environmental domains. Mean scores for Pakistani were lower in all four domains when compared to Portugal [18] and Malawi [19]. Compared with Kuwait [20], Pakistani domain scores were higher in the psychological and environmental domain. Moreover in comparing the study results with Skevingtone et al’s WHO 23 – country report we found the QOL in Pakistani subjects was lower than the total WHO subjects in the physical, psychological and environmental health domains, while the mean score for the social relationship was higher than the WHO’s score [5].

The final model after multiple linear regression analysis showed that socioeconomic status and social capital were statistically significant in all the four domains of QOL, type of residence was significant in physical, psychological and environmental health domains. Furthermore, gender and age were significant in physical, psychological and social relation health domains. Presence of disease was only significant in physical and psychological health domains.

The QOL of women was found to be lower than that of men in the physical and psychological and social relationship health domains. These results were consistent with further studies, in which women reported poorer QOL than men in both physical and psychological domains in Iranian general population [3]. This finding was not found in a study conducted in Japan which showed better QOL scores in women [4]. The prominent reasons behind less QOL scores in Pakistani women were due to marriage at the early age, male baby gender preference of male baby, higher prevalence of depression and other mental disorders and lower marital satisfaction rate [21–23].

Rural residence was negatively associated with decreased QOL scores in physical health, psychological health and environmental health domain. Similar findings were also reported in Brazil with higher QOL score in all domains among those who lived in urban areas [24]. Contrastingly, another study from Pakistan in hemodialysis patients showed better QOL in the rural area as compared to urban in physical and environmental health domains [25]. Similarly, a study in Nepal showed significantly decreased scores in physical and social relationship domains among those who lived in urban residence [26]. Furthermore, no statistical difference in all domains was found in a study conducted in Thailand by using HADS and WHO BREF Questioner [27]. The decreased QOL scores among rural areas in our study might be due to the lack of facilities in terms of better living conditions, access to hospitals, transport, quality education, security, physical mobility, entertainment and shopping centers.

Our study finding revealed that domains of quality of life were also influenced by age, as age increases, QOL scores significantly decreases. Our finding supports previous reports on QOL with elders the most affected group [28, 29]. The reasons by which our results were becoming more authentic that emotional sensitivity increases with age because of loneliness, increasing tendency towards age related diseases and continuous hard work to give the best standard of living to their families. Moreover, anxiety, social pressure, economic issues, loss of important family members or friends and psychological problems were also common reasons in Pakistani society [29]. Furthermore, younger age groups have less responsibilities on their shoulders as most of the times they are accommodated and supported by their parents.

Socioeconomic status was also significantly correlated to all the four domains of QOL in Pakistani population. People with higher socioeconomic levels reported better QOL score. Our study findings are in accordance to those of the Chinese, Portuguese and Brazilian general population [2 ,18, 30]. Being a developing country, the effect of low socioeconomic status is more likely to be felt by the more vulnerable people of the society and hence result in lower QOL.

The relationships of health status and QOL in previous studies suggest that presence of diseases like diabetes, hypertension, depressive disorder, functional disabilities etc. could lead to poor QOL. Current illness was found as a predictor of poor QOL in these studies [31–34]. People having no disease have better QOL in both physical and psychological health domains, while no significant relationship was observed in the remaining two domains among disease and non-disease groups.

The present study also establishes the highly significant association between QOL and social capital in physical, psychological, social relationship and environmental health domains.

Consistent with prior studies conducted on AIDS patients and the elderly population [35–37], our analysis showed that higher level of social capital is associated with better QOL. High social capital is linked with better QOL scores and this was evident in our study where all domains were statistically significant.

Although we found no significant association between family structures (joint/nuclear) and QOL of the participants in this study, other studies found it an important determinant of QOL [38–41]. However, various studies conducted in India have reported that there is no significant difference in various QOL domains among joint and nuclear family systems [25, 42].

The joint family system is a tradition in Pakistani society since partition from India in 1947. However, in the present era, the trend is moving towards nuclear families. The possible reason behind this non-significant relationship may be that participants in our sample have adopted to their family type in this long period. They prefer to live in the same family structure in which they were living [in their childhood]. They are competent of fulfilling all types of needs of its members. It helps them to initiate and to sustain growth, and to be an origin of care, safety, and inspiration. Secondly, because of strong connections to their roots and norms, a person from the nuclear family is used to visit his/her [joint] family house quite frequently in order to make available himself/herself whenever needed. Thirdly, Abbottabad is a cultural district without having not much difference in the lifestyle and socioeconomic status of joint and nuclear family systems, which may be one of the reasons for not having a significant difference in QOL scores.

The strengths of this study includes the local data collection from all UCs of district Abbottabad according to their population, making it possible to understand the factors which commonly affect the QOL. Our study was community based and one of the first of its kind, as most of the previous studies evaluated QOL focusing on diseased population. The strength of the study is that it employed the validated and standardized WHOQOL-BREF to measure quality of life. The instrument has been validated in the Urdu language among the Pakistani population [7].

Furthermore, to the best of our knowledge, this is the first study describing the QOL of the general Pakistani population by using WHOQOL-BREF.

This data reflects the experiences of one district of KPK province. Among limitations are the need for caution regarding the generalizability of the present study as diverse tribes with a different culture reside in District Abbottabad, so the study sample may not be representative of the remaining provinces of the country. Furthermore, this is a cross-sectional study therefore it is limited, to assessing the association, rather than causality between QOL and other factors.

## Conclusions

To the best of our knowledge, this study is the first in Pakistani general population demonstrating the association between socio-demographic variables and QOL domains. Overall, subjective QOL was found to be low in our population and greatly varied by socio-demographic variables. Increasing age, having average or lower socioeconomic status and living in the rural area were found to be a strong predictor of poorer QOL in all domains, while total social capital score had a positive effect on Pakistani QOL scores. There is a need to carry out the QOL studies on a continuing and regular basis to track the trend and direction.

## Additional file

Additional file 1: All 52 Union councils along with population and division of houses from both types of families from district Abbott bad, Pakistan. (DOCX 14 kb)

## Abbreviations

CI: Confidence Interval
LL: Lower limit
QOL: Quality of life
R: Reference
SC-IQ: Social Capital integrated Questioners
SD: standard deviation
SES: Socioeconomic status
Std. Err: Standard error
TUMS: Tehran University of Medical Sciences
UL: Upper limit
WHOQOL: World health organization quality of life
β: Beta coefficient

## References

[1] World Health Organization (1948). WHO constitution.

[2] Cruz LN, Polanczyk CA, Camey SA, Hoffmann JF, Fleck MP. Quality of life in Brazil: normative values for the Whoqol-bref in a southern general population sample. Qual Life Res. 2011. 10.1007/s11136-011-9845-3. 10.1007/s11136-011-9845-3 21279448

[3] Nedjat S, Naieni KH, Mohammad K, Majdzadeh R, Montazeri A. Quality of life among an Iranian general population sample using the World Health Organization’s quality of life instrument (WHOQOL-BREF). Int J Public Health. 2011. 10.1007/s00038-010-0174-. 10.1007/s00038-010-0174-z 20680655

[4] Ohaeri JU, Awadalla AW, Gado OM (2009). Subjective quality of life in a nationwide sample of Kuwaiti subjects using the short version of the WHO quality of life instrument. Soc Psychiatry Psychiatr Epidemiol.

[5] Skevington SM, Lotfy M, O'Connell KA. The World Health Organization's WHOQOL-BREF quality of life assessment: psychometric properties and results of the international field trial. A report from the WHOQOL group. Qual Life Res. 2004. 10.1023/B:QURE.0000018486.91360.00. 10.1023/B:QURE.0000018486.91360.00 15085902

[6] Bonomi AE, Patrick DL, Bushnell DM, Martin M. Validation of the United States’ version of the World Health Organization quality of life (WHOQOL) instrument. J Clin Epidemiol. 2000. 10.1016/S0895-4356(99)00123-7. 10.1016/s0895-4356(99)00123-7 10693897

[7] Saqib Lodhi F, Raza O, Montazeri A, Nedjat S, Yaseri M, Holakouie-Naieni K. Psychometric properties of the Urdu version of the World Health Organization's quality of life questionnaire (WHOQOL-BREF). Med J Islam Repub Iran. 2017; 10.14196/mjiri.31.129. PMC6014800 29951429

[8] Brajsa-Zganec A, Merkas M, Sverko I (2011). Quality of life and leisure activities: how do leisure activities contribute to subjective well-being?. Soc Indic Res.

[9] Wahl AK, Rustøen T, Hanestad BR, Lerdal A, Moum T (2004). Quality of life in the general Norwegian population, measured by the quality of life scale (QOLS-N). Qual Life Res.

[10] Earthquake Reconstruction and Rehabilitation Authority Prime Minister’s Secretariat I. District Profile - Abbottabad. 2007, p. vii.

[11] Haq M, Mustafa U, Ahmad I (2007). Household’s willingness to pay for safe drinking water: a case study of Abbottabad district. Pakistan Development Review.

[12] NIPS II. Pakistan Demographic and Health Survey 2012–13. Islamabad. 2013. http://www.nips.org.pk/abstract_files/PDHS%20Final%20Report%20as%20of%20Jan%2022-2014.pdf. Accessed 30 Nov 2017.

[13] World Health Organization (1997). WHOQOL: measuring quality of life: the development of the World Health Organization quality of life instrument (WHOQOL).

[14] Harper A, Power M (1998). Development of the World Health Organisation WHOQOL-BREF quality of life assessment. Psychol Med.

[15] Grootaert C, Narayan D, Nyhan Jones V, Woolcock M: Measuring Social Capital: An Integrated Questionnaire (SC-IQ). Washington D.C.: The International Bank for Reconstruction and Development/The World Bank; 2004.

[16] Zaslavsky AM. Using hierarchical models to attribute sources of variation in consumer assessments of health care. Stat Med. 2007. 10.1002/sim.2808. 10.1002/sim.2808 17221833

[17] Goldstein H. Multilevel statistical models. 2nd ed. London: Edward Arnold; 1995.

[18] Patrício B, Jesus LM, Cruice M, Hall A. Quality of life predictors and normative data. Soc Indic Res. 2014. 10.1007/s11205-013-0559-5.

[19] Colbourn T, Masache G, Skordis-Worrall J. Development, reliability and validity of the Chichewa WHOQOL-BREF in adults in Lilongwe, Malawi. BMC Res Notes. 2012. 10.1186/1756-0500-5-346. 10.1186/1756-0500-5-346 PMC3483688 22759784

[20] Ohaeri JU, Awadalla AW, Gado OM. Subjective quality of life in a nationwide sample of Kuwaiti subjects using the short version of the WHO quality of life instrument. Soc Psychiatry Psychiatr Epidemiol. 2009. 10.1007/s00127-008-0477-z. 10.1007/s00127-008-0477-z 19037572

[21] Qadir F, Khan MM, Medhin G, Prince M. Male gender preference, female gender disadvantage as risk factors for psychological morbidity in Pakistani women of childbearing age-a life course perspective. BMC Public Health. 2011; 10.1186/1471-2458-11-745. PMC3195096 21958069

[22] Mirza I, Jenkins R. Risk factors, prevalence, and treatment of anxiety and depressive disorders in Pakistan: systematic review. BMJ. 2004. 10.1136/bmj.328.7443.794. 10.1136/bmj.328.7443.794 PMC383372 15070634

[23] Mumford DB, Saeed K, Ahmad I, Latif S, Mubbashar MH. Stress and psychiatric disorder in rural Punjab. A community survey. Br J Psychiatry. 1997. 10.1192/bjp.170.5.473. 10.1192/bjp.170.5.473 9307700

[24] dos Santos Tavares DM, Fernandes Bolina A, Aparecida Dias F, dos Santos Ferreira PC, José HV (2014). Quality of life of elderly. Comparison between urban and rural areas. Invest Educ Enferm.

[25] Anees M, Malik MR, Abbasi T, Nasir Z, Hussain Y, Ibrahim M. Demographic factors affecting quality of life of hemodialysis patients–Lahore, Pakistan. Pak J Med Sci. 2014. 10.12669/pjms.305.5239. 10.12669/pjms.305.5239 PMC4163245 25225539

[26] Mishra SR, Sharma A, Bhandari PM, Bhochhibhoya S, Thapa K. Depression and health-related quality of life among patients with type 2 diabetes mellitus: a cross-sectional study in Nepal. PLoS One. 2015; 10.1371/journal.pone.0141385. PMC4658137 26599008

[27] Nuttaset Manimmanakorn M, Vichiansiri R, Nuntharuksa C, Permsirivanich W, Vilai Kuptniratsaikul M (2008). Quality of life after stroke rehabilitation among urban vs. rural patients in Thailand. J Med Assoc Thai.

[28] Kaur G, Kaur D, Verma P (2016). Elderly Women’s quality of life in a conflict border area of India. Int J Adv Res Manag Soc Sci.

[29] Mohyuddin A, Rehman I (2016). Psychological factors of aging in Pakistan. Indian J Health Well Being.

[30] Xia P, Li N, Hau K-T, Liu C, Lu Y. Quality of life of Chinese urban community residents: a psychometric study of the mainland Chinese version of the WHOQOL-BREF. BMC Med Res Methodol. 2012; 10.1186/1471-2288-12-37. PMC3364902 22452994

[31] Gureje O, Kola L, Afolabi E (2007). Epidemiology of major depressive disorder in the Ibadan Studyof ageing. Lancet.

[32] Bekibele CO, Gureje O (2008). Impact of self-reported visual impairment on quality of life in the Ibadan study of ageing. Br J Ophthalmol.

[33] Akinpelu A, Maruf F, Adegoke B (2006). Validation of a Yoruba translation of the World Health Organization’s quality of life scale--short form among stroke survivors in Southwest Nigeria. Afr J Med Med Sci.

[34] Hu Y, Mak JN, Wong YW, Leong JC, Luk KD (2008). Quality of life of traumatic spinal cord injured patients in Hong Kong. J Rehabil Med.

[35] Campos ACV, Borges CM, Leles CR, Lucas SD, Ferreira EF. Social capital and quality of life in adolescent apprentices in Brazil: An exploratory study. 2013; DOI:10.4236/health.2013.56128.

[36] Ma Y, Qin X, Chen RL, Li NN, Chen R, Hu Z (2012). Impact of individual-level social capital on quality of life among AIDS patients in China. PLoS One.

[37] Nilsson J, Masudrana AM, Naharkabir Z (2006). Social capital and quality of life in old age results from a cross-sectional studying rural Bangladesh. J Age Health.

[38] Hernández RL, Aranda BE, Ramírez MTG (2009). Depression and quality of life for women in single-parent and nuclear families. Span J Psychol.

[39] Turagabeci AR, Nakamura K, Kizuki M, Takano T. Family structure and health, how companionship acts as a buffer against ill health. Health Qual Life Outcomes. 2007; 10.1186/1477-7525-5-61. PMC2234394 18036211

[40] Munaf S, Siddiqui B. Relationship of post-natal depression with life and marital satisfaction and its comparison in joint and nuclear family system. Procedia Soc Behav Sci. 2013; 10.1016/j.sbspro.2013.06.636.

[41] Kumar G, Majumdar A. Quality of life (QOL) and its associated factors using WHOQOL-BREF among elderly in urban Puducherry, India. J Clin Diagn Res. 2014. 10.7860/JCDR/2014/6996.3917. 10.7860/JCDR/2014/6996.3917 PMC3939587 24596723

[42] Pawar PR, Adsul R (2015). Influence of gender and nature of family on psychological well-being among adolescents. Indian J Health Well being..

