# Supplementary material for: Assessing the quality of life among Pakistani general population and their associated factors by using the World Health Organization’s quality of life instrument (WHOQOL-BREF): a population based cross-sectional study
Source: Health Qual Life Outcomes. 2019 Jan 14;17:9. doi: 10.1186/s12955-018-1065-x (PMC6332637; doi:10.1186/s12955-018-1065-x)
Supplement: Supplementary file 1 — All 52 Union councils along with population and division of houses from both types of families from district Abbott bad, Pakistan. (DOCX 14 kb) [file 12955_2018_1065_MOESM1_ESM.docx]

Appendix 1

| **No.** | **Unions** |  | **Nuclear** | **Joint** |
| --- | --- | --- | --- | --- |
| 1 | **DALOLA** | 22428 | 23 | 23 |
| 2 | **BOI** | 20175 | 21 | 21 |
| 3 | **KUKMONG** | 12867 | 13 | 13 |
| 4 | **NAMBAL** | 16819 | 17 | 17 |
| 5 | **PATTAN KALAN** | 19125 | 19 | 19 |
| 6 | **BAKOT** | 27475 | 28 | 28 |
| 7 | **BEROTE KALAN** | 30868 | 31 | 31 |
| 8 | **PALAK** | 22543 | 23 | 23 |
| 9 | **NAGRIL BALA** | 15321 | 16 | 16 |
| 10 | **NATHIA GALI** | 19820 | 20 | 20 |
| 11 | **NAMLI MAIRA** | 13164 | 13 | 13 |
| 12 | **BAGNOTAR** | 11098 | 11 | 11 |
| 13 | **BEERANGALI** | 14631 | 15 | 15 |
| 14 | **SARBANA** | 13461 | 14 | 14 |
| 15 | **PHALKOT** | 11602 | 12 | 12 |
| 16 | **BAGH** | 15712 | 16 | 16 |
| 17 | **KUTHWAL** | 13847 | 14 | 14 |
| 18 | **BANDA PIR KHAN** | 30124 | 31 | 31 |
| 19 | **BALDHERI** | 17558 | 18 | 18 |
| 20 | **MIRPUR(50%R)** | 22268 | 23 | 23 |
| 21 | **KAKUL** | 20661 | 21 | 21 |
| 22 | **DHAMTOUR** | 21945 | 22 | 22 |
| 23 | **SHEIKHULBANDI (50%R)** | 21589 | 22 | 22 |
| 24 | **SALHAD(50%R)** | 25171 | 26 | 26 |
| 25 | **JHANGI(75%R)** | 31122 | 32 | 32 |
| 26 | **PAWA** | 21747 | 22 | 22 |
| 27 | **CHAMHAD** | 21151 | 22 | 22 |
| 28 | **PIND KARGU KHAN** | 21070 | 21 | 21 |
| 29 | **KUTHIALA** | 13821 | 14 | 14 |
| 30 | **JARRAL** | 8242 | 8 | 8 |
| 31 | **SHERWAN** | 18165 | 18 | 18 |
| 32 | **MALIKPURA (URBAN)** | 19616 | 20 | 20 |
| 33 | **CENTRAL  ABBOTTABAD** | 17914 | 18 | 18 |
| 34 | **KEHAL (URBAN)** | 18967 | 19 | 19 |
| 35 | **NAWANSHEHR  (URBAN)** | 22987 | 23 | 23 |
| 36 | **GHARI PHULGRAN** | 24247 | 25 | 25 |
| 37 | **BANDI ATTAI KHAN** | 16439 | 17 | 17 |
| 38 | **JHANGRA (25%U)** | 28040 | 29 | 29 |
| 39 | **MAJOHAN** | 18427 | 19 | 19 |
| 40 | **LANGRIAL** | 14775 | 15 | 15 |
| 41 | **NARA** | 18619 | 19 | 19 |
| 42 | **DEWAL MANAL** | 12934 | 13 | 13 |
| 43 | **GOREENI** | 18413 | 19 | 19 |
| 44 | **PHALLAH** | 22542 | 23 | 23 |
| 45 | **LORA** | 13923 | 14 | 14 |
| 46 | **LANGRA** | 19824 | 20 | 20 |
| 47 | **HAVELIAN (URBAN)** | 24405 | 25 | 25 |
| 48 | **SEER GHARBI** | 10556 | 11 | 11 |
| 49 | **TAJWAL** | 11589 | 12 | 12 |
| 50 | **NAGRI TOTIAL** | 11105 | 11 | 11 |
| 51 | **SEER SHARQI  BHATTIAN** | 9819 | 10 | 10 |
|  |  |  |  |  |
|  | **GRAND TOTAL DISTRICT ABBOTTABAD)** | **950731** | **968** | **968** |
